# The resilience of two professionalized departmental health insurance units during the COVID-19 pandemic in Senegal

**DOI:** 10.7189/jogh.10.020394

**Published:** 2020-12

**Authors:** Ndeye Bineta Mbow, Ibrahima Senghor, Valéry Ridde

**Affiliations:** 1 Departmental Health Insurance Unit (UDAM) of Foundiougne, Senegal; 2 Departmental Health Insurance Unit (UDAM) of Koungheul, Senegal; 3 Centre Population et Développement (Ceped), Institut de recherche pour le développement (IRD) et Université de Paris, Inserm, France; 4 Institut de Santé et Développement, Université Cheikh Anta Diop, Dakar, Sénégal

**Figure:**
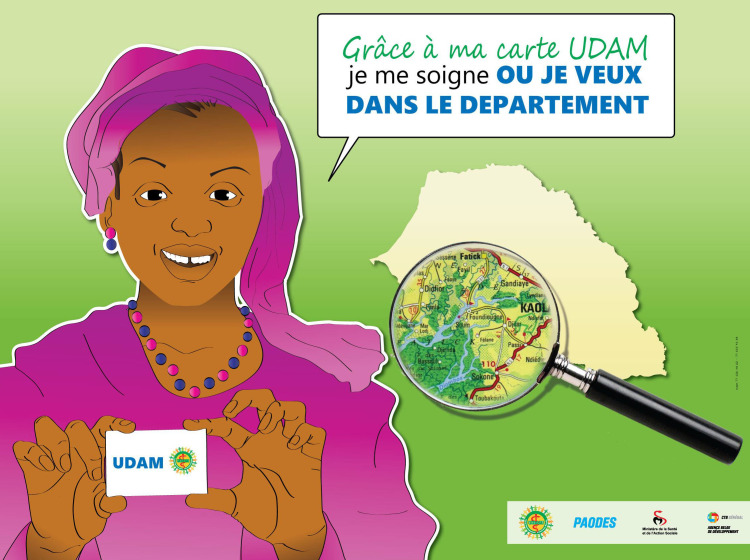
Photo: from Paodes/Enabel, used with permission.

In its desire to extend health care coverage, Senegal has, in recent years, embarked on several health financing strategies [1,2], at the risk of some fragmentation [3]. The strategic plan for the development of universal health coverage (UHC) 2013-2017 aims to achieve the objective of covering at least 75% of the population at risk of illness by 2021. After introducing community-based health insurance [4,5] and the various user fees exemption intervention for certain categories of people (people aged 60 and over, children under 5, the poor) or certain services (caesarean sections, HIV treatment, dialysis) [6-9], Senegal has tested two models for extending health risk coverage. The first relates to the decentralization of health insurance (DECAM) with the creation of community-based health insurance at the communal level. At the end of 2019, a national assessment estimates that 80% of community-based health insurance policies have fewer than 500 beneficiaries [10]. The second is large-scale professionalized health insurance with, for the moment, two departmental health insurance units (UDAM) in Koungheul and Foundiougne [11]. From 2013 to 2017, the formulation and implementation of these two UDAMs have been organized by the Ministry of Health and Social Action with Belgian Technical Cooperation (currently Enabel), through its Care Supply and Demand Support Project (PAODES).

The UDAM system adopted by the authorities is a model of large-scale insurance units based on the following principles:

- An operational unit at department level with centralized management;
- A professionalization of its organization with 1 general manager, 1 financial manager, 1 medical advisor, 1 to 3 administrative assistants, 2 to 4 premium collectors, and few other support staff;
- Financing combining the contribution of the population and subsidies from the State and/or external partners;
- *A posteriori* control of the quality of health services offered to UDAM beneficiaries by the health facilities;
- A transparent, uniform, flat-rate pricing system subsidized by UDAM at the higher level (Health Centre);
- An integration of local and regional authorities in the decision-making bodies (Board of Directors) facilitating the appropriation of the model;
- Representation of populations from grassroots community organizations by local government branches.

## EFFECTIVE UDAMS IN THE CONTEXT OF A PANDEMIC IN SENEGAL

The results of the UDAMs were capitalized on, and a National Forum on UHC was organized [12]. It resulted in the publication of a collective work [11], several scientific papers in international conferences, and numerous policy briefs.

**Figure 1:**
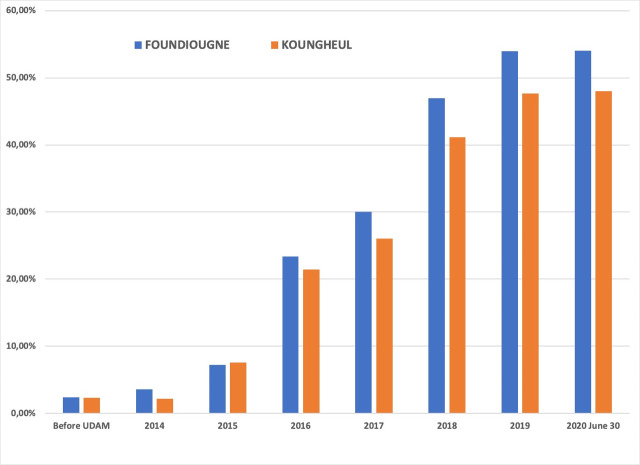
Evolution of the penetration rate of the two UDAMs.

Enabel's support (PAODES) ended in June 2017, meaning that the UDAMs had to organize themselves without this international technical and financial support. In spite of these challenges, and unlike community-based health insurance that generally do not recover from the cessation of support from partners [13], the UDAMs have coped. Their financial viability is still significant, their performance indicators have not fallen, and they have even made significant progress, according to their penetration rates (**Figure 1**). At the national level, it is estimated that the penetration rate is 33% in 2019, including the two UDAMs, and that 69% of beneficiaries are up to date with their annual contribution [10].

**Figure 2:**
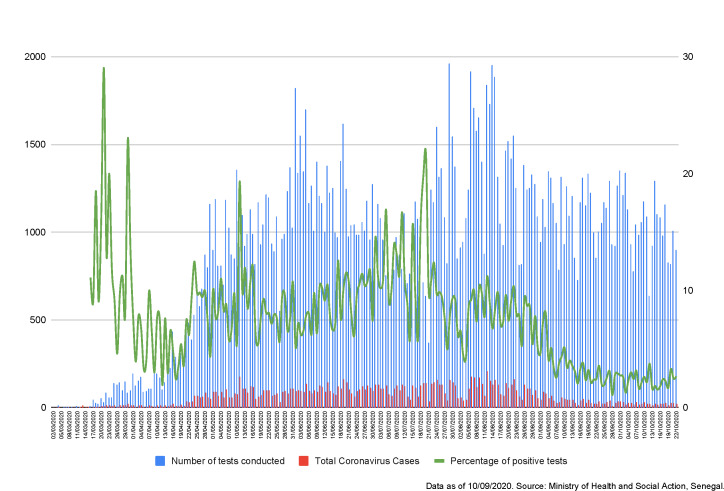
Evolution of COVID-19 cases and testing in Senegal by June 30, 2020. Data as of 7 September 2020. Source: Ministry of Health and Social Action, Senegal.

The UDAMs have been able to adapt, innovate, and organize themselves to deploy strategies underpinned by *a priori* sustainability processes that are favourable to their sustainability [14]. However, Senegal, like all countries in the world, was hit at the beginning of March 2020 by the SARS-CoV-2 (COVID-19) pandemic [15]. **Figure 2** shows the evolution of cases of COVID-19 since the beginning of the pandemic. As of Sept 7th, 2020, 14 014 cases and 291 deaths had been recorded (https://www.covid19afrique.com).

Although the number of cases and deaths remains much lower than elsewhere in the world and a plateau seems to have been reached, the measures taken by the State to contain the pandemic have had an impact on the operation of the UDAMs. While a few studies on the resilience of insurance and health systems have been started in Europe [16], we think it is useful to examine the resilience of UDAM in Senegal [17]. This is an initial exploratory reflexive paper, in particular to respond to the call for greater reflexivity by those involved in population health interventions [18] in order to share our first ideas and to prepare, at a later stage, an empirical research part of the UNISSAHEL program (https://www.unissahel.org).

## CHALLENGES AND ADAPTATIONS OF UDAM ACTIVITIES IN A PANDEMIC CONTEXT

Our analysis revealed at least four major challenges that UDAMs have had to face since the beginning of the pandemic by implementing innovative solutions.

### Challenge 1: Participating responsibly in the fight against the pandemic

Like everyone else in Senegal, the UDAMs felt immediately and fully involved in the health response to the pandemic. But it was not a question of responding to it by putting its employees at excessive risk. Thus, all the agents were trained by the district chief doctors in the public health issues of the disease, both at the clinical level and in COVID-19 prevention actions. The UDAMs were also directly involved in the response. Thus, their directors are members of the departmental epidemic management committee and the multisectoral communal pandemic committees. In Koungheul, UDAM's technical staff accompanied the community relays and the commune's student volunteers in their home visits to raise awareness among the population on the respect of barrier gestures. In Foundiougne, Red Cross volunteers came to support UDAM in the organization of safe reception (taking of temperature, hand washing, etc.) for beneficiaries in UDAM's building. The UDAM also financially supported the departmental response plan by granting a budget of 550 000 F CFA (€840) to Foundiougne and 1 500 000 F CFA (€2300) to Koungheul. This made it possible, for example, to purchase disinfectant products.

### Challenge 2: Maintain contribution collection

The UDAMs live largely on the membership premiums. In a context where travel was restricted and direct contact between people was subject to physical distancing, they had to be innovative without participating in the spread of the coronavirus. For the renewal of membership fees, electronic payments were promoted after informing members and, when necessary, by subsidizing mailing costs. New members were advised to send photos and other necessary documents via WhatsApp. The collectors relied on the UDAM focal points in the villages to optimize the collection of premiums and reduce the amount of travel required. In Foundiougne, for members with more than 10 beneficiaries and who did not have, in the difficult economic conditions during the pandemic, the possibility of renewing insurance for all, the strategy adopted for the UDAM consisted in asking them to follow the family's enrolment order registered in the management software. This is a form of tacit constraint to prevent the head of household from selecting only the sickest members of the household when renewing the contribution, to the detriment of the others. The UDAMs here face the classic dilemma of the trade-off between moral hazard and financial viability in order to guarantee the sustainability of the services offered to its members.

### Challenge 3: To continue caring for beneficiaries in health facilities

During the period when it was forbidden to move between communes, it was not possible to provide the documents enabling the indigent (families benefiting from the State family security grant entitling them to a free state-subsidized premium to the UDAM) to renew their membership. In order to guarantee permanent access to health care for these poorest people, specific correspondence has been sent to all chief doctors. Likewise, when a patient who is a member of a UDAM is referred, he or she must first come and collect a letter of referral from the departmental head office. Thus, it was possible to request this letter at the more local level of the district. For people who have to go to the hospital, the UDAMs sent a direct care letter by e-mail. In addition, since the follow-up of complaints at the local level has been reduced by limiting the travel of UDAM agents, it is during the monthly coordination meetings at the district level and in the presence of the nurses in charge of the health facilities that the difficulties encountered by patients have been addressed.

### Challenge 4: Ensuring payment of invoices in a context of government delays

Like all health facilities in the country, UDAMs face a significant delay by the Senegalese State in reimbursing fees associated with exemptions or subsidies for the payment of contributions for certain categories of people. In addition to this delay of nearly two years in payment, which is exacerbated in the context of the pandemic, there is also a reduction in the use of health care and a reduction in financial resources for health centres and private pharmacies. In Koungheul department, it was the primary health posts that pre-financed emergency medical evacuations to the hospital and the UDAM reimbursed them at the end of the month after the monthly bill was sent. However, in the context of the pandemic accentuated by the delay in reimbursement by the State, UDAM had difficulty in honouring this monthly reimbursement, which led some structures to require beneficiaries to pay evacuation costs, even though their membership of UDAM entitles them to this service free of charge. Faced with this situation, UDAM undertook to reimburse monthly emergency medical evacuations to health posts in order to prevent UDAM beneficiaries from losing something they had acquired. Similarly, in view of the delay in reimbursement of State subsidies, the reimbursement of medicines in private pharmacies had been temporarily suspended, and this was reflected in the introduction of a system for reimbursing the part attributable to UDAM to the beneficiaries at the end of each quarter (the Foundiougne pharmacy did not cease to provide services or incur debts to the health facilities). After the payment of part of the subsidies by the State, the UDAM of Koungheul settled the debts owed to private pharmacies and the population now continues to pay only 50% of the price of the prescription.

## CONCLUSION

While the UDAMs were able to cope with the cessation of support from their international technical and financial partner in mid-2017, the arrival of the pandemic in March 2020 is a new long-lasting test. The UDAMs have been innovative in order to face the new challenges generated by the pandemic at the local and national level in their organizational routine. We have described these challenges and the solutions implemented, which seem to demonstrate the UDAM's ability to react in order to guarantee their members the services they expect. This is at the heart of the resilience of an insurance organization in the context of UHC [16,17,19,20]. Research is now essential to organize in order to understand the foundations of the actions taken to guarantee these services, the challenges of their implementation and, above all, their effectiveness. However, given the resilience shown by UDAM and its staff when their main partner left in 2017, we have reason to believe that the pandemic will not be able to break this dynamic in favour of UHC in Senegal.

## References

[1] Deville C, Hane F, Ridde V, Touré L. La Couverture universelle en santé au Sahel: la situation au Mali et au Sénégal en 2018. Paris; 2018 p. 38. (Working Papers du CEPED (40)).

[2] Daff BM Diouf S Diop ESM Mano Y Nakamura R Sy MM Reforms for financial protection schemes towards universal health coverage, Senegal. Bull World Health Organ. 2020;98:100-8. 10.2471/BLT.19.239665 32015580 PMC6986231

[3] Mladovsky P Fragmentation by design: universal health coverage policies as governmentality in Senegal. Soc Sci Med. 2020;260:113153. 10.1016/j.socscimed.2020.113153 32663695

[4] Jütting J Do Community-based Health Insurance Schemes Improve Poor People’s Access to Health Care? Evidence From Rural Senegal. World Dev. 2004;32:273-88.

[5] Alenda-Demoutiez J Boidin B Community-based mutual health organisations in Senegal: a specific form of social and solidarity economy? Rev Soc Econ. 2019;77:417-41. 10.1080/00346764.2018.1555646

[6] Mladovsky P Ba M Removing user fees for health services: A multi-epistemological perspective on access inequities in Senegal. Soc Sci Med. 2017;188:91-9. 10.1016/j.socscimed.2017.07.002 28734964

[7] Paul E Deville C Bodson O Sambiéni NE Thiam I Bourgeois M How is equity approached in universal health coverage? An analysis of global and country policy documents in Benin and Senegal. Int J Equity Health. 2019;18:195. 10.1186/s12939-019-1089-9 31847877 PMC6915934

[8] Deville C, Escot F, Ridde V, Touré L. Les processus d’identification des plus pauvres à l’épreuve du terrain: une comparaison Bénin-Mali-Sénégal. In Roskilde University, Denmark; 2018. Available: http://hdl.handle.net/2268/226060. Accessed: 7 September 2020.

[9] Ndiaye S Le fonds d’équité au Sénégal: analyse des mécanismes de la couverture maladie des indigents et de ses perspectives pour la couverture maladie universelle. Afr Dev. 2017;42:9-31.

[10] Ministère du développement communautaire, de l’équité sociale et territoriale. Analyse situationnelle des organisations mutualistes à base communautaire dans le cadre de la Couverture Maladie Universelle de janvier 2017 à septembre 2019. Dakar; 2020 p. 112.

[11] Bossyns P, Ladrière F, Ridde V. Une assurance maladie à grande échelle pour le secteur informel en Afrique subsaharienne Six ans d’expérience au Sénégal rural 2012 – 2017. Studies in Health Services Organisation&Policy, 34, 2018. Antwerp: ITGPress; 2018.

[12] Ridde V Dagenais C What we have learnt (so far) about deliberative dialogue for evidence-based policymaking in West Africa. BMJ Glob Health. 2017;2:e000432. 10.1136/bmjgh-2017-000432 29259821 PMC5728265

[13] Waelkens M-P Coppieters Y Laokri S Criel B An in-depth investigation of the causes of persistent low membership of community-based health insurance: a case study of the mutual health organisation of Dar Naïm, Mauritania. BMC Health Serv Res. 2017;17:535. 10.1186/s12913-017-2419-5 28784123 PMC5545852

[14] Ridde V Pluye P Queuille L Evaluer la pérennité des programmes de santé publique: un outil et son application en Haïti. Rev Epidemiol Sante Publique. 2006;54:421-31. 10.1016/S0398-7620(06)76740-2 17149163

[15] Bonnet E. Bodson oriane, Marcis FL, Faye A, Sambieni E, Fournet F, et al. The COVID-19 Pandemic in Francophone West Africa: From the First Cases to Responses in Seven Countries. In Review; 2020 Aug. Available from: https://www.researchsquare.com/article/rs-50526/v1. Accessed: 2 October 2020. 10.1186/s12889-021-11529-7 PMC8327893 34340668

[16] Legido-Quigley H Mateos-García JT Campos VR Gea-Sánchez M Muntaner C McKee M The resilience of the Spanish health system against the COVID-19 pandemic. Lancet Public Health. 2020;5:e251. 10.1016/S2468-2667(20)30060-8 32199083 PMC7104264

[17] Turenne CP Gautier L Degroote S Guillard E Chabrol F Ridde V Conceptual analysis of health systems resilience: A scoping review. Soc Sci Med. 2019;232:168-80. 10.1016/j.socscimed.2019.04.020 31100697

[18] Tremblay M-C Parent A-A Reflexivity in PHIR: Let’s have a reflexive talk! Can J Public Health. 2014;105:e221-3. 10.17269/cjph.105.4438 25165844 PMC6972395

[19] Ridde V Benmarhnia T Bonnet E Bottger C Cloos P Dagenais C Climate change, migration and health systems resilience: Need for interdisciplinary research. F1000Res. 2019;8:22. 10.12688/f1000research.17559.2 32983410 PMC7506192

[20] Blanchet K, Diaconu K, Witter S. Understanding the Resilience of Health Systems. In: Bozorgmehr K, Roberts B, Razum O, Biddle L, editors. Health Policy and Systems Responses to Forced Migration. Cham: Springer International Publishing; 2020.

